# Preparation of Novel Epoxy Resins Bearing Phthalazinone Moiety and Their Application as High-Temperature Adhesives

**DOI:** 10.3390/polym10070708

**Published:** 2018-06-26

**Authors:** Liwei Wang, Jinyan Wang, Yu Qi, Fengfeng Zhang, Zhihuan Weng, Xigao Jian

**Affiliations:** 1 Liaoning High Performance Resin Engineering Research Center, Department of Polymer Science & Materials, Dalian University of Technology, Dalian 116024, China; jhwlw2008@163.com (L.W.); wangjinyan@dlut.edu.cn (J.W.); qiyudy001@mail.dlut.edu.cn (Y.Q.); Zhangfeng0908@126.com (F.Z.); 2 PetroChina Co., Ltd., Jilin Petrochemical Branch, Jilin 132022, China

**Keywords:** poly(aryl ether), phthalazinone, epoxy adhesive, high-temperature resistant

## Abstract

Most polymer-based adhesives exhibit some degree of degradation at temperatures above 200 °C, and so there is a need for the development of adhesives that can be used at high temperatures. A series of poly(phthalazinone ether nitrile sulfone ketone)s terminated with epoxy (E-PPENSK) and amine (A-PPENSK) groups have been prepared, which have been used as precursors can be applied for high-temperature resistant epoxy adhesives. The structured of these E-PPENSK (epoxy resin) and A-PPENSK (curing agent) components have been characterized by ^1^H nuclear magnetic resonance (NMR) and Fourier transform–infrared spectroscopy (FT–IR) studies, with the effects of molecular weights and molar ratios on the gel content of their polymers being determined. Cured epoxy resins derived from E-PPENSK and A-PPENSK showed good thermal stability, with an optimal resin retaining 95% of its weight at 484 °C, which gave a char yield of 62%. This adhesive was found to exhibit good mechanical strength, with a single-lap adhesive joint (A-3000/E-6000) exhibiting a shear strength of 48.7 MPa. Heating this adhesive at 450 °C for 1 h afforded a polymer that still exhibited good shear strength of 17.8 MPa, indicating that these adhesives are potentially good candidates for high-temperature applications.

## 1. Introduction

Adhesives are vital components of a large number of devices used by the automotive, aerospace, electronics, and naval industries [1], with many of these applications requiring adhesives that can function under severe environmental conditions, such as high temperatures. Consequently, there is significant demand for new polymer-based adhesives that can withstand high temperatures [2,3], with polymer-based adhesives used for high-temperature applications requiring a high glass transition temperature (*T*_g_). Epoxy resins that exhibit high thermal stabilities are considered to be good candidates as adhesives, with these resins often displaying good strength and ductility at high temperatures [3,4,5,6,7].

Epoxy resins possess many desirable properties, such as high tensile strength and modulus, excellent chemical and solvent resistance, high dimensionality and thermal stability, good creep resistance, and excellent adhesive properties [8,9,10,11]. High temperature formulations of epoxy adhesives have recently been reported, with Baneaet al. [5] reporting that single lap joints bonded with epoxy adhesives can function at high temperatures. They found that the tensile strength of these adhesives dropped from 68 MPa at rt to 45 MPa at 100 °C, with a relatively high value of 6.5 MP still being retained at 150 °C. Chen et al. [12] reported that epoxy resin could be used to enhance the properties of soy-based adhesives, including their gluability, water resistance, solid content, viscosities and thermal stabilities. Indeed, the wet shear strength of *Pinus massoniana* plywood bonded with 30% defatted soy flour (DSF) mass epoxy resin-modified soy-based adhesives was found tobe sufficient to meet Chinese National Standards for exterior plywood. Recently, Lahouar et al. [13] reported the mechanical behavior of adhesive anchors at high temperatures, describing that the resin–glass transition was responsible for their lower fire resistance at higher temperatures. Interestingly, it was found that heating these types of epoxy chemical anchors resulted in an improvement in their mechanical properties, due to heat activation of post-curing reaction pathways.

Thermoplastics have been investigated as additives for epoxy resins with the aim of improving their thermal properties for high-performance engineering applications [14]. These toughened epoxy resins are produced through blending with various thermoplastics, such as polysulfone [15], poly(ether sulfone) [16,17], poly(ether imide) [18,19], poly(amide-amidic acid) [20], poly(ether ether ketone) [21,22]. We have previously prepared a series of engineering phthalazinone-based thermoplastics that exhibit high mechanical strengths and good thermal properties [23]. For example, a novel thermoplastic co-poly(phthalazinone ether sulfone) with a *T*_g_ of 265 °C was blended with the diglycidyl ether of bisphenol A epoxy resin (DGEBA) to improve the thermal stability of an epoxy resin [24]. However, although the addition of engineering thermoplastics can improve the thermal stability of epoxy resins, the morphology and extent of phase separation can adversely affect the mechanical properties of these cured blend systems [17,18]. 

This study describes the synthesis of high-temperature resistant epoxy resin and evaluates their performance as adhesive for high temperature applications. Poly(phthalazinone ether nitrile sulfone ketone)s terminated with epoxy and amine group have been used as precursors to prepare adhesives. The wholly aromatic twisted non-coplanar structure gifts the polymers outstanding thermal stability and excellent mechanical properties as well as good solubility. The adhesives can be obtained easily via blending the two polymers followed by thermal treating without any other additives, which may result in morphology and phase separation. Moreover, the properties of the adhesives can be tuned in a large range via changing the dosage and molecular weight of the polymers. Then the structures and thermal stabilities of the resulting polymers were characterized by various technologies, including Fourier transform–infrared spectroscopy (FT-IR), ^1^H nuclear magnetic resonance (NMR), differential scanning calorimetry (DSC), and thermogravimetric (TG) analysis. The prepared adhesive with high thermal stability and shear strength were discussed. 

## 2. Materials and Methods

### 2.1. Materials

1,2-Dihydro-4-(4-hydroxyphenyl)-1-(2*H*)-phthalazinone (DHPZ) was kindly supplied by Dalian Polymer Materials Co., Ltd. (Dalian, China) and was used after further purification in *N*,*N*-dimethylacetamide. 2,6-difluorobenzonitrile (DFBN), 4,4′-dichlorodiphenyl sulfone (DCS), di-*p*-fluorophenylketone (DFK), 4-aminophenol, epichlorohydrin, anhydrous potassium carbonate and dimethyl sulfoxide (DMSO) were purchased from Aladdin Chemical Reagent Co., Ltd. (Shanghai, China) and used as received. 

### 2.2. Preparation of Polymers

**Scheme 1:**
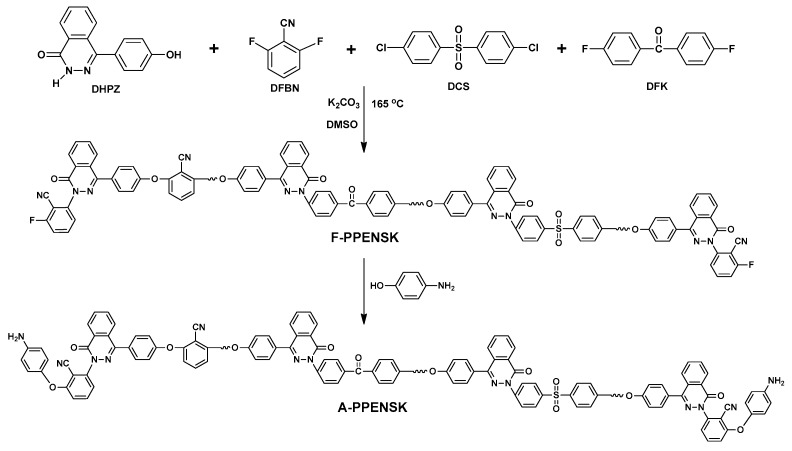
Reaction route for preparing amine-terminated polymer A-PPENSK.

**Synthesis of poly(phthalazinone ether nitrile sulfone ketone)s terminated with amine (A-PPENSK).** The A-PPENSK was synthesized by terminating the fluorine-containing poly(phthalazinone ether nitrile sulfone ketone) (F-PPENSK) with 4-aminophenol (Scheme 1). Firstly, the F-PPENSK was prepared by solution polycondensation reaction based on DHPZ, DFBN, DCS and DFK. In a typical procedure, a stirred solution of anhydrous K_2_CO_3_ (3.864 g, 28 mmol), DMSO (80 mL) and toluene (120 mL) were addedto DHPZ (4.76 g, 20 mmol), DFBN (1.112 g, 8 mmol), DCS (1.716 g, 6 mmol) and DFK (1.308 g, 6 mmol) under a nitrogen atmosphere was refluxed at 140 °C for 3 h. Water and residual toluene were then removed by distillation (atmospheric pressure) and the temperature of the reaction mixture was then raised to 165 °C to allow for polymerization to occur over a period of 6–8 h. The mixture was then poured slowly into stirred hot water, resulting in precipitation of a white polymer that was purified by extraction with boiling distilled water to remove residual DMSO and K_2_CO_3_. The resultant crude solid was then dissolved in chloroform to afford a solution containing 15% polymer content, that was filtered, with the filtrate then poured into ethanol to afford a white flocculent copolymer that was dried under vacuum at 120 °C for 24 h. The yield of F-PPENSK produced in this process was around 90%.

0.6 g of 4-aminophenol in 5 mL DMSO was added dropwise to a stirred solution of 3 g of F-PPENSK, 0.8 g of potassium carbonate, 20 mL of DMSO and 30 mL of toluene over a 30 min period. The reaction mixture was then heated at 140 °C for 2 h, followed by distillation of water and toluene from the system. The reaction was then heated to 165 °C for 2 h, before post-reaction work-up using hot water and recrystallization from chloroform to afford A-PPENSK in around 90% yield. This general procedure was used to prepare A-PPENSKs with different molecular weights of 3000, 6000, 8000 and 12,000, that were classified as A-3000, A-6000, A-8000 and A-12000, respectively.

**Synthesis of poly(phthalazinone ether nitrile sulfone ketone)s terminated with epoxy (E-PPENSK).** A mixture of DHPZ (4.76 g, 20 mmol), DFBN (0.834 g, 6 mmol), DCS (1.716 g, 6 mmol), DFK (1.308 g, 6 mmol), ground anhydrous K_2_CO_3_ (3.864 g, 28 mmol), DMSO (80 mL) and toluene (120 mL) were refluxed at 140 °C until all the water had been removed through azeotropic distillation with toluene. The reaction mixture was then heated at 165 °C for 6–8 h, before being cooled to 100 °C, ground anhydrous K_2_CO_3_ (3.45 g, 25 mmol) and epichlorohydrin (3.7 g, 40 mmol) added, and the reaction heated at 100 °C for a further 12 h. The resultant solution was then poured into hot water, and the crude solid washed three times with hot water, before being dried and purifiedby precipitation from CHCl_3_. The solid obtained was dried under vacuum at 80 °C for 24 h to afford E-PPENSKs with different molecular weights 3000, 6000, 8000 and 12,000, that were classified as E-3000, E-6000, E-8000 and E-12000, respectively.

**Curing procedure for A-PPENSK/E-PPENSK mixture.** A-PPENSK (0.5 g, A-3000), E-PPENSK (1 g, E-3000) and CHCl_3_ (3 mL) were combined to afford a viscous solution that was cast onto a glass slide, with solvent then removed at 80 °C under vacuum to afford a product that was cured in an air-circulating oven at 230 °C for 1 h, 260 °C for 1 h, 290 °C for 2 h and then 300 °C for 1 h.

### 2.3. Characterization

^1^H NMR (400 MHz) was obtained in deuterated chloroform (CHCl_3_-d) using a Varian INOVA400 spectrometer (Palo Alto, CA, USA) with tetramehylsilane as internal reference. Infrared measurements were performed on a Thermo Nicolet Nexus 470 Fourier transform–infrared (FT–IR, Waltham, MA, USA) spectrometer. Gel permeation chromatography (GPC) analyses were carried out on an Agilent PL-50 (Palo Alto, CA, USA) using polystyrene as standard and CHCl_3_ as the eluent. DSC measurements were obtained using a Mettler DSC1 differential scanning calorimeter (Zurich, Switzerland) under a flow of nitrogen at a heating rate of 10 °C/min from 25–350 °C. Thermogravimetric analysis (TGA) curves were determined using a Mettler TGA1 thermal gravimetric analysis instrument (Zurich, Switzerland) under a flow of nitrogen (50 mL/min) at a heating rate of 10 °C/min over a temperature range of 30–800 °C. The strength tests of adhesives were performed on specimens made of hot-dip galvanized steel sheet with the dimension of 100 × 25 × 2 mm. The tests were performed on single-lap adhesive joints, and the length of lap was 15 mm. Adhesive joints were produced under programmed temperature with a load set to 0.05 MPa. Following the curing and seasoning process, the strength tests were conducted on an INSTRON 5567A cupping machine (Boston, MA, USA) to determine the hot-dip galvanized steel sheet adhesive joints shear strength with the displacement rate of 5 mm·min^−1^, and at least five specimens were tested for every condition. The gel content of the cured epoxy adhesive was determined by putting a known weight of adhesive sample (W_i_) into CHCl_3_ solvent for 24 h. Then the solid sample was removed from the solvent and the residual was dried in an oven to achieve constant weight (W_f_). Gel content was calculated by using the following equation: gel content (%) = (W_f_/W_i_) × 100%. Where, W_f_ and W_i_ are the final and initial weight of the sample, respectively.

## 3. Results and Discussion

### 3.1. Synthesis of Epoxy Resin Based on Poly(Phthalazinone Ether Nitrile Sulfone Ketone)s

**Scheme 2:**
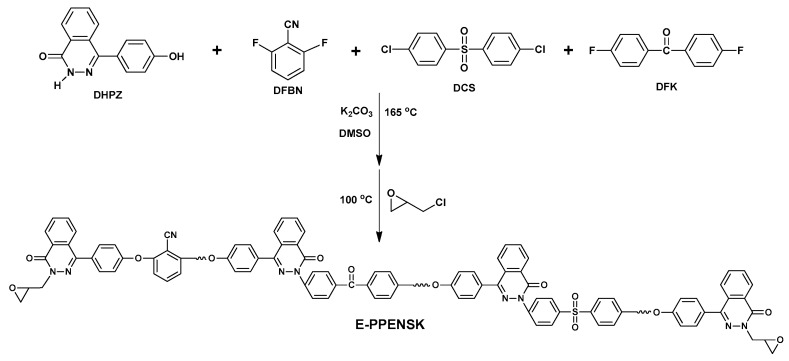
Reaction route for preparing epoxy-terminated polymer E-PPENSK.

We, and others have previously prepared a series of engineering thermoplastics containing non-coplanar twisted phthalazinone moietiesthat exhibit reasonable solubilities and highthermal stabilities. These polymers have been widely used as matrix resins in advanced composites for applications in aerospace and microelectronic industries, and as components for membrane technology [25,26,27,28]. This study describes the preparation of polymers from nucleophilic attack of 1,2-dihydro-4-(4-hydroxyphenyl)-1-(2*H*)-phthalazinone (DHPZ) displacement on chlorine (or fluorine) substituents of 2,6-difluorobenzonitrile (DFBN), 4,4′-dichlorodiphenyl sulfone (DCS) and di-*p*-fluorophenylketone (DFK). The molecular weights of these copolymers have been controlled by varying the molar ratio of DHPZ to DFBN, with prepolymerization times, monomer concentrations, and feeding mode being used to control the molecular weight of the final copolymer. A DHPZ-based polymer terminated with amino groups (A-PPENSK) has been prepared in two steps (Scheme 1), whilst an epoxy polymer (E-PPENSK) has been produced using a one-pot procedure (Scheme 2).

**Scheme 3:**
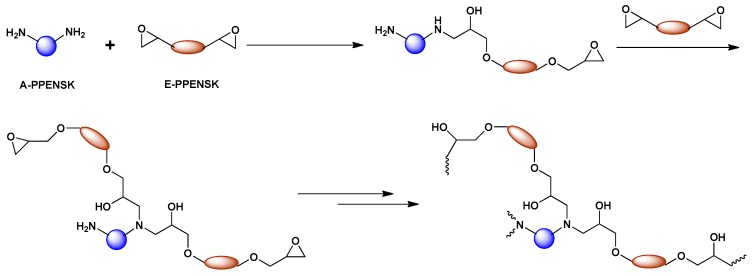
Curing mechanism of epoxy resin E-PPENSK with A-PPENSK as curing agent.

**Figure 1:**
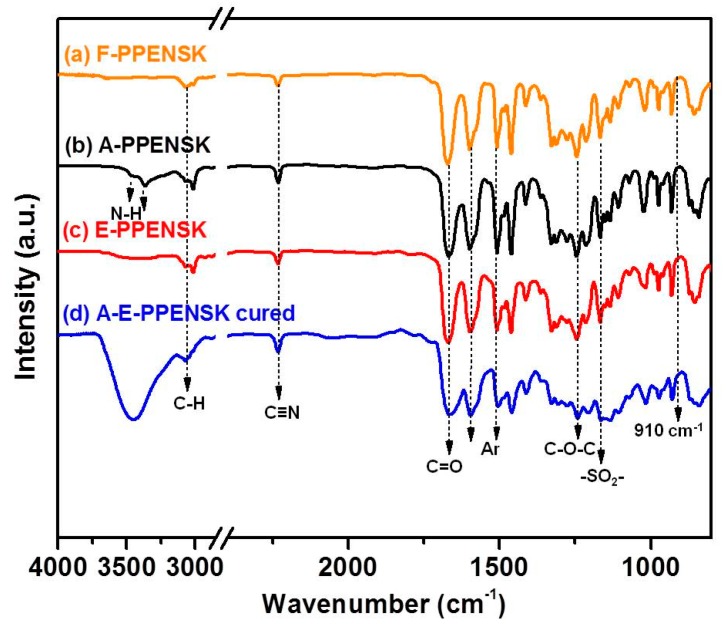
Fourier transform–infrared (FT–IR) spectra of various polymers.

**Figure 2:**
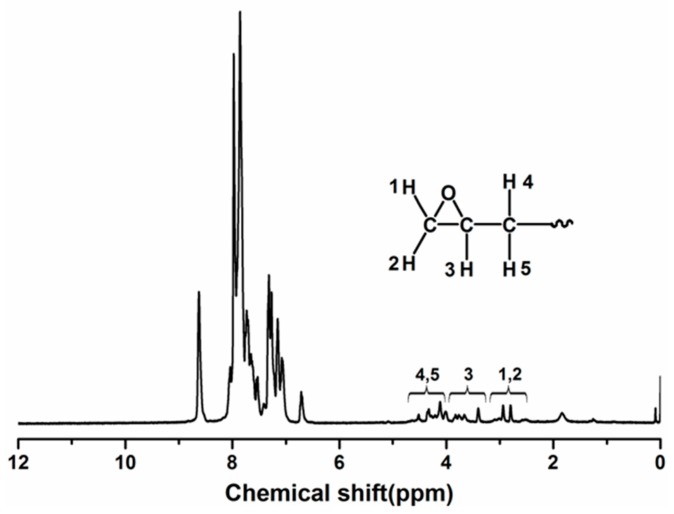
^1^H nuclear magnetic resonance (NMR) spectrum of polymer E-PPENSK terminated with epoxy group.

The structures of as-prepared polymers were identified by FT–IR. As shown in Figure 1a, the bands at 3066 and 3016 cm^−1^, 2230 cm^−1^, 1662 cm^−1^, 1594 and 1506 cm^−1^, 1244 cm^−1^, 1167 cm^−1^ were assigned to the vibration of aromatic C-H, C≡N, C=O, benzene skeleton, aromatic ether, and sulfonyl group, respectively. These results clearly indicated that the structure of polymer F-PPENSK as shown in Scheme 1 had been obtained successfully. The spectra of polymers terminated with amine and epoxy groups were really similar with that of A-PPENSK, as they had the same main chain structure, except the terminal groups. The two peaks at 3452 and 3376 cm^−1^ can be considered as evidence for the presence of a terminal amine group in A-PPENSK. However, the peak around 910 cm^−1^ attributed to the epoxy group was not detected in the IR spectrum of E-PPENSK (Figure 1b), it is possible that the characteristic peak was overlapped by the nearby peak as the content of terminal epoxy group was low in the whole polymer. Fortunately, with the help of liquid proton NMR, which is a more sensitive characterization technique compared with FT–IR, the peaks attribute to terminal epoxy group can be clearly identified as shown in Figure 2, although it is not easy to discriminate all of the peaks in the low field due to the complicated aromatic structure of the polymer. The adhesive can be easily prepared by blending these two copolymers A-PPENSK and E-PPENSK together, followed by thermal curing. The mechanism of polymerization that occurs when epoxy resinsare cured by primary aminesis well documented [29], with Scheme 3 illustrating the steps that occur for the polymerization reactions described in this study. The FT–IR spectra of resins that were produced (Figure 1c) indicate that the core structure of the polymer was maintained during the curing process, which is key tothe thermal stability of the resulting adhesives.

The solubilities of as-prepared polymers were tested in various organic solvents by dissolving 10 mg of polymers in 1 mL of solvent at room (and elevated) temperatures, as shown in Table 1. Aromatic engineering plastics generally exhibit poor solubility in organic solvents; however, A-PPENSK and E-PPENSK were soluble in common polar aprotic solvents (NMR, DMF, DMAc and CHCl_3_) at room temperatures. The excellent solubilities of these polymers in organic solvents can be assigned to the presence of their twisted non-coplanar phthalazinone structures, which increase chain-packing distances and reduce intermolecular interactions between their macromolecular chains [30].

### 3.2. The Effect of Formula of Adhesive on Gel Content

**Figure 3:**
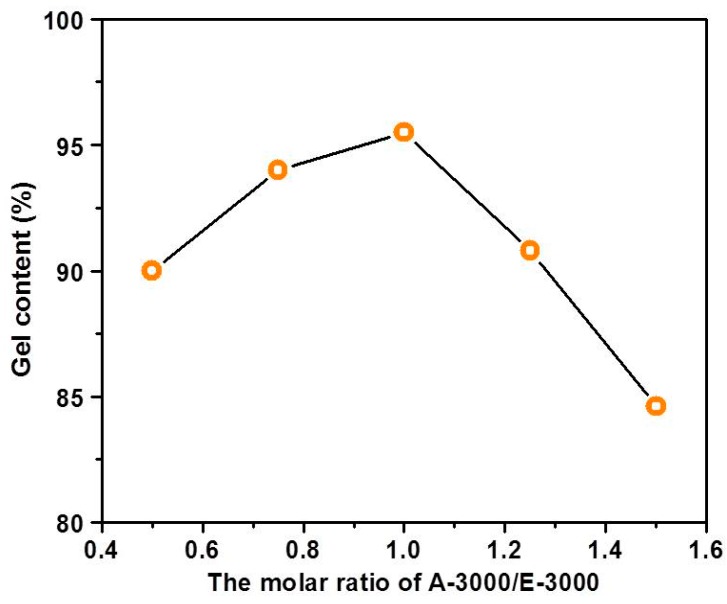
The effect of molar ratio of the two selected polymers on gel content.

**Figure 4:**
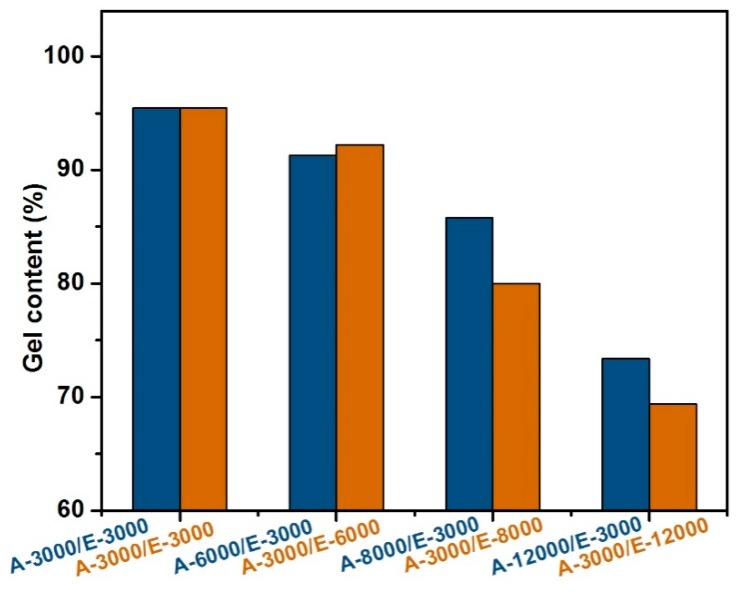
The effect of molecular weight of the two selected polymers on gel content.

The gel content of the cured adhesives in CHCl_3_ were studied over a 24 h period, with A-PPENSK (A-3000) and E-PPENSK (E-3000) of 3000 g/mol molecular weight chosen to determine the effect of their molar ratios on gel content (see Figure 3). Increasing the ratio of A-3000 to a 50% limit resulted in a corresponding increase in gel content, with A-3000 content levels greater than 50% leading to a decrease in gel content. A 1:1 ratio of the A-PPENSK and E-PPENSK polymers was chosen to determine the effect of molecular weight on adhesive performance (Figure 4). Adhesives derived from polymers with the lowest molecular weights had the highest gel content, with increased gel content being caused by more crosslinked structures in the cured adhesive [31]. Conversely, increasing monomer molecular weight results in the presence of longer chains that leads to the segmental mobility and overall density of the epoxy and amine groups being decreased, thus resulting in lower amounts of gel formation.

### 3.3. The Effects of Formula and Curing Procedure on Shear Strength

**Figure 5:**
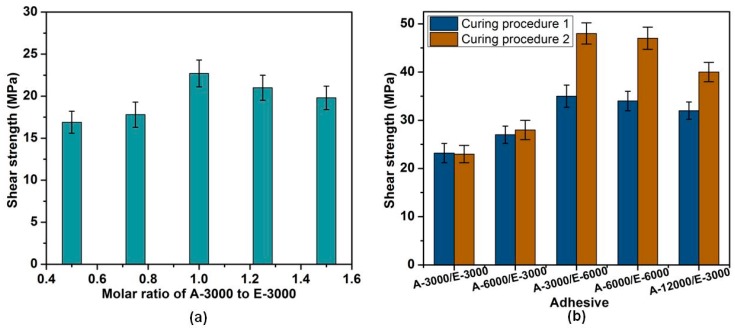
The shear strength of adhesives cured with (**a**) various molar ratios of A-3000 to E-3000, and (**b**) various procedures, the molar ratio of the two selected polymers was 1:1.

Hot-dip galvanized steel sheet adhesive joints prepared from different polymer compositions were cured at different temperatures using two different curing procedures. The first curing procedure involved heating at 200 °C for 1 h, 230 °C for 1 h, 260 °C for 2 h and 290 °C for 1 h, whilst the second curing procedure involved heating at 230 °C for 1 h, 260 °C for 1 h, 290 °C for 2 h and 300 °C for 1 h. Polymers prepared using the first curing were used to determine the effect of varying the molar ratio of A-3000 to E-3000 on shear strength (Figure 5a). Curing an epoxy resin using a primary amine as curing agent normally requires an optimal ratio of two epoxy groups and one primary amino group [29,32]. However, it was found that the adhesive with the highest shear strength values were produced when a 1:1 molar ratio of A-3000 to E-3000 was used for curing. It is likely that this deviation from an ideal 2:1 stoichiometry is due to the mobility of the primary amine groups ofa macromolecular curing agent being lower than for low molecular weight primary amines, which reduces the overall efficiency of the curing process so that an excess of A-PPENSK is required for complete curing (Figure 5a).

Adhesives derived from A-3000/E-6000 monomers had the highest shear strength (regardless of the curing process employed), thus indicating that good adhesive performance requires optimal matching of the molecular weights of the two polymer components (Figure 5b). Low molecular weight adhesives normally exhibit low viscosities with their fluid mobile sidechains resulting in low cohesive forces that produce poor shear strengths. However, when the molecular weight of an adhesive becomes too great, then its cohesive energy is increased so that its bonding strength is compromised. Therefore, polymers with moderate molecular weights are considered the best choice for preparing adhesives with good binding properties. Differences in the shear strengths of low molecular weight adhesives cured at different temperatures were not as great as those observed for high molecular weight adhesives. This is because low molecular weight adhesives are easily cured, even at relatively low temperatures, whilst higher molecular weight adhesives require high curing temperatures for complete curing to occur. The excellent joint shear strength observed for these adhesives is attributed to their intrinsic toughness that is produced by the higher interfacial strength of its adhesively bonded structure. The second curing procedure was found to afford the highest degree of curing, which gave good interfacial bonding between the adhesive and its substrate, leading to greater shear strength in the jointed structure [33,34]. One point that also should be noted is that, when the molecular weight of E-PPENSK was fixed, for example at 3000, the shear strength of the adhesive can be enhanced with increasing the molecular weight of A-PPENSK in the range of 3000–12,000. Based on these results, the second curing procedure was chosen as curing condition for further study.

### 3.4. Thermal Stability of Adhesive

**Figure 6:**
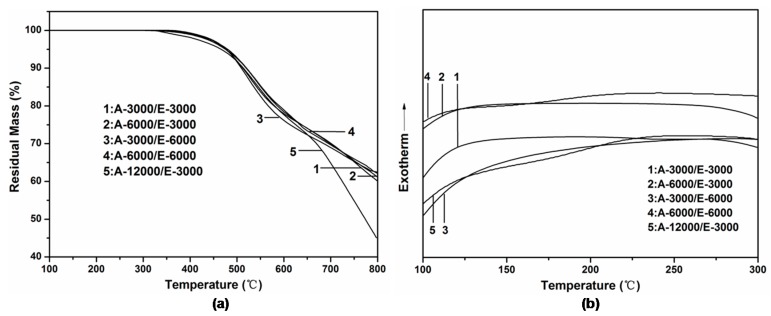
(**a**) Thermogravimetric analysis (TGA) traces of adhesive; (**b**) differential scanning calorimetry (DSC) curves of adhesives.

The thermal properties of the adhesives were investigated over a temperature range of 30–800 °C in a TGA chamber under a nitrogen atmosphere (Figure 6a). Cured A-3000/E-6000 adhesives showed the highest thermal stability, retaining 95% of its weight at 484 °C, with a char yield of 62%. The glass transition temperature (*T*_g_) of these polymers could not be detected within a temperature range of 30–300 °C, which indicates that the *T*_g_ values of this adhesive must be greater than 300 °C.

### 3.5. The Mechanical Properties after Treated under High Temperature

**Figure 7:**
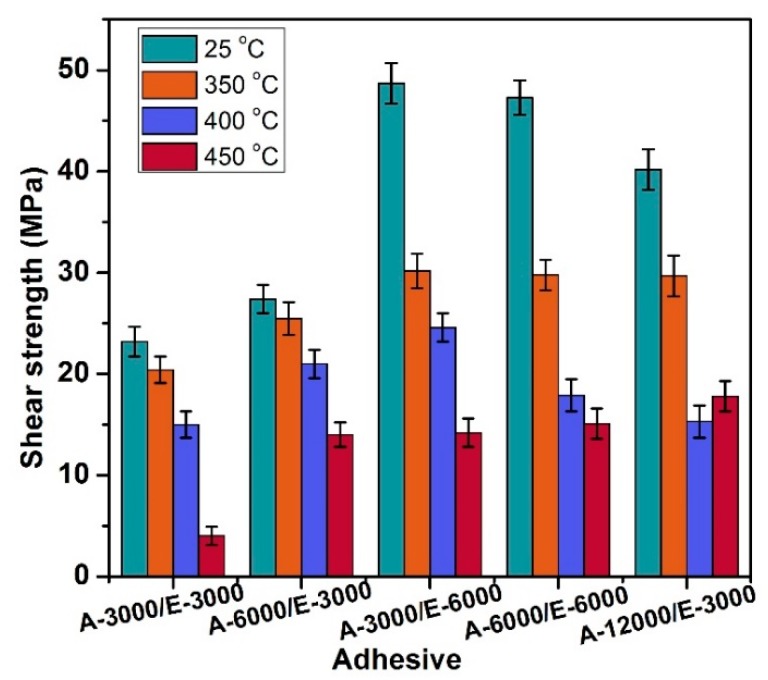
The shear strength of samples treated at different temperatures.

Based on above TGA data, we can see that the temperature at maximum weight loss for all of the tested samples was around 540 °C, to further test the application of adhesives at high temperature, the shear strength was detected after the sample was treated at 350 °C, 400 °C and 450 °C for 1 h, respectively. As shown in Figure 7, for all of the samples, the shear strength was declined gradually with increasing the treatment temperature. Before heat treatment, the highest shear strength was obtained in the sample of A-3000/E-6000 with the value of 48.7 MPa, with gave reduced values of 30.2 and 24.6 MPa being measured after treatment at 350 and 400 °C for 1 h, respectively. The decline of shear strength was caused by the enhancement of the movability of the molecular chain and the viscosity of the resin with increasing the treatment temperature. The highest shear strength at 450 °C after 1 h was obtained using an adhesive composed of a mixture of A-12000/E-3000 which gave a value of 17.8 MPa. These shear strength results clearly indicate that these adhesives exhibit good mechanical and thermal resistance properties. However, it is clear that adhesives with low molecular weight showed worse heat resistance, for example, after being treated at 450 °C for 1 h, the shear strength of the adhesive with the formula A-3000/E-3000 was reduced to 4.0 MPa. This also can be explained by the low cohesive force in the adhesive with low molecular weight. Finally, the properties of resulting adhesives were compared with previous work. The key parameters including *T*_g_ and shear strength at room temperature are summarized in Table 2, and it is clear that our adhesive with optimal formula showed better performance than those reported in literature.

## 4. Conclusions

This study has shown that poly(phthalazinone ether nitrile sulfone ketone)s terminated with epoxy (E-PPENSK) and amine (A-PPENSK) can be used to prepare thermally resistant adhesives that exhibit high glass transition temperatures and excellent shear strength properties. The presence of non-coplanar phthalazinone structures in these polymers confers thermal stability and improves their solubility in organic solvents, which facilitates adhesive production. Thermal curing of a mixture of A-3000 and E-6000 monomers resulted in an adhesive that exhibited 5% weight loss at 484 °C, with a char yield of 62% at 800 °C and a *T*_g_ value of greater than 300 °C. These properties indicate that this class of adhesives is likely to prove useful for high-temperature applications in a wide range of industries.

## Figures and Tables

**Table 1:** Solubilityof the polymers in various solvents ^a^.

Polymers	Solvents ^b^							
	NMP	DMF	DMSO	DMAc	THF	CHCl_3_	Sulfolane	Acetone
A-PPENSK	++	++	+	++	-	++	+	-
E-PPENSK	++	++	+	++	-	++	+	-
Cured A-E-PPENSK	-	-	-	-	-	-	-	-

^a^ Solubility: ++, soluble at room temperature; +, soluble on heating; -, insoluble. ^b^ NMP, *N*-methyl pyrrolidone; DMF, *N*,*N*-dimethylformamide; DMSO, dimethyl sulfoxide; DMAc, *N*,*N*-dimethylacetamide; THF, tetrahydrofuran.

**Table 2:** Comparison the presented results with literature data.

Epoxy Adhesive	*T*_g_ (°C)	Shear Strength at Room Temperature (MPa)	Ref.
A-PPENSK/E-PPENSK	>300	48.7	This work
XN1244	155	31.6	[5]
Cordierite	170	45	[35]
LARC-TPI	~236	41.4	[36]
TMSPMA	~170	32.5	[37]
